# Design recommendations for active games

**DOI:** 10.3389/fdgth.2022.814226

**Published:** 2022-09-16

**Authors:** Pamela N. Martinez

**Affiliations:** Learning Games Lab, New Mexico State University, Las Cruces, NM, United States

**Keywords:** active games, exergames, physical activity, video games, health, design

## Abstract

Active gaming is a form of video gaming that requires full body motion or varying degrees of physical activity to play a game. While active gaming has regained momentum, the design and specific components of active games that make them engaging are limited. This study identifies, analyzes, and categorizes specific design mechanics and features used in active games. It answers the question: Which, if any, game mechanics and features can a panel of experts in the academic, health, and game industry agree on as valuable and impactful to the construction of successful and engaging active games? Using a Delphi study, nine experts answered questions related to active gaming. They reached an agreement on 20 of the 21 inquiries regarding game design focused on motivation, social influence, and flow. Their feedback offers recommendations on the design of future active games and identifies emerging trends. This study shares their notes and translates the findings into specific recommendations for developers on the design of active games. The field of active gaming has matured; there are pockets of experts in design, research, and implementation. Active gaming has maintained continuity; however, player enthusiasm and engagement in these types of games are consistently an issue. Through better game design and newer types of active games, players’ interest will persist. These guidelines can inform developers working with newer technologies, such as mobile devices, enhanced game consoles, and virtual and augmented reality platforms, to create active games that inspire gamers to play.

## 1. Introduction

Childhood obesity has tripled in children aged 6–11 years in the last 30 years from 7% to nearly 21% and quadrupled from 5% to 22% in adolescents aged 12–19 years in the same period (1, 2). Obesity continues to be an issue for today's youth, with an increased risk for cardiovascular diseases, diabetes, stroke, and osteoarthritis in young adults, much younger than previous generations. Healthy lifestyles such as increased physical activity can help lower these risks (3). Lifestyle choices are influenced by many factors such as society, families, communities, schools, physical environments, and media. Media consumption and increased use of personal technologies like mobile phones, tablets, computers, and gaming consoles are often blamed for the rise of sedentary lifestyles.

Active games can aid obesity prevention and other related health issues (4–7). Additional positive outcomes include physical, psychosocial, cognitive, and academic (5, 8). Serious game designers, researchers, and policymakers view games as a means to create social impact since games can be explicitly designed to improve quality of life by addressing issues such as economics, the environment, and health (9–11). Often, game play results directly impact real life by creating positive behavioral change (9, 12, 13). Gaming technologies have changed dramatically and are slowly being recognized as possible aids in obesity prevention. Consumers spent 25.62 billion dollars in the gaming industry in 2019 (14). Gaming *via* consoles, computers, and mobile devices has become increasingly physical with the breakthrough of sensory-based technology.

The Entertainment Software Association reports that 74% of American households play video games (15). Parents recognize the positive impacts of gaming, benefits such as mental stimulation, support in education, an increase in family time, and social connections with friends (15, 16). With more than half of American households playing video games, it is conceivable to extend outreach beyond families to schools, after-school programs, community centers, and other social groups to enhance physical activity and learning.

Active game researchers, healthcare practitioners, and game developers have unique perspectives on what makes games successful. Active gaming is still a relatively new phenomenon, especially with the rapidly changing technologies in consoles and mobile devices. Motion sensors such as gyroscopes, accelerometers, compasses, and barometers have significantly changed the potential of games to require physical activity. Despite the increase of research in active gaming, research providing recommendations on how design influences player motivation is limited. Additionally, active game research often focuses on the impact of a single intervention rather than an overview of methods used across the field. Finally, the active gaming field includes academic researchers, game designers, and implementation experts with experience in using active games with a wide variety of users. Although active gaming continues to develop, there is still only a small scale of experts in the design, research, and implementation of active games.

Individuals with expertise in research, practice, and development each have a unique perspective on how the design of active games influences motivation. The purpose of this study was to fill an information gap by giving experts ways to discuss the design of active games with colleagues who have strengths in each of these areas. While experts in this field are known to each other, they rarely meet in person due mainly to their geographic location and diverse backgrounds (17). Nine experts from the academic, health, and the game industries participated in surveys on active gaming. They reached an agreement on 20 of the 21 inquiries regarding game design focused on motivation, social influence, and flow. Their input offers recommendations and identifies new trends to aid in designing future active games. These recommendations can help developers working with modern technologies to construct engaging games that encourage players to play.

## 2. Methodology

A Delphi methodology was used to seek consensus or majority agreement among this panel of experts with diverse professional backgrounds. The Delphi structure allows informed individuals from different disciplines or specialties to contribute information and opinions to a study to address an issue, problem, forecasting, or examination that benefits from a wider scope of knowledge than that of a lone individual or specialization (17). This technique is completed through an integrative process of communication, mainly surveys. This study acknowledges that each respondent works primarily in one of three different roles: who are using or have used active games with various populations in a multitude of settings, who design active games, or who research impacts.

Through this Delphi study, experts evaluated statements about specific types of motivation, social influence, and flow related to active gaming. Each question included an open response space for respondents to clarify or justify their selection opportunity for open-ended input. After the first round of questions, the answers were reviewed for consensus among the respondents. When answers to a question did not reflect a consensus among respondents, the question was reissued in Round 2, with samples of previous answers shown so that participants could reflect and have the opportunity to change their perspective if necessary. Responses were reviewed, coded, and analyzed for either further inquiry in Round 2 or the final conclusion. All retired questions were shared with participants in Round 2, noting the results of Round 1. Eventually, both rounds were coded and analyzed to identify common themes and recommendations.

The research was approved by Pepperdine University’s Institutional Review Board (Supplementary Material), and all participants provided their consent.

### 2.1. Reliability and validity

To ensure reliability and validity, a pilot test was administered to a small group of three participants who were not preselected for the main study. The pilot test group consisted of games and educational technology professionals at the game development studio with research expertise in active games. After the pilot test was administered, the pilot test participants were asked to analyze the usability of the test to determine if the survey items, including directions, were easy to read and comprehend. These suggested changes were implemented before the final research project began.

### 2.2. Subjects and sampling

Fourteen expert participants for this study were identified as leaders in the field and reflected three different perspectives: academics, practitioners, and game designers. The selection criterion for the participants is based on their visibility, publications, and referrals. Twelve participants were chosen, with nine completing the survey.

Academics, the first category, include six experts who actively research active games in various environments. Academic researchers publish active game articles in various journals in fields such as medicine, physical education, and games for health; have incorporated clinical studies in their research; and have become authorities in this field.

Two experts were practitioners within medical or community health professions who have purposefully used active games in communities, schools, or medical practices. The medical and community health practitioners were chosen based on their extensive community outreach work with obesity prevention and physical fitness and their extensive use of active games. This group of experts either prescribes or conducts interventions with a wide variety such audiences and locations such as medical centers, county extension service outreach, schools, and exergaming facilities.

The final area of expertise, game design, provided one reviewer who has created games for health as well as other genres and is a professional in the industry. Game designers were chosen for their expertise in game design and are distinguished in health-related games. The designers are the target audience for implementing the findings of this study. They are familiar with game mechanics and content specific to health games.

### 2.3. Instrumentation: the Delphi survey

The survey design was a statement, with a 5-point Likert scale ranging from strongly agree to strongly disagree and no opinion. Each question included an open response space for respondents to clarify or justify their selection opportunity for open-ended input.

#### 2.3.1. Design regarding motivation

Motivation is what prompts people to engage in some type of activity. In gaming, what motivates gamers to play comes from many different influences and perspectives. This section centers around motivation and how it may influence active game design.

#### 2.3.2. Design regarding social influences

Video gaming, in most regards, has become a very social activity. Sociability in gaming is accomplished through online communities and in real life; it also influences teamwork and competition. This section centers around social influences and how they may influence active game design.

#### 2.3.3. Design regarding flow

People are happiest when they are at their optimal level of concentration (18). An absorption so deep the world around ceases to exist (18). This effortless state of engagement is referred to as “flow” (18). This section centers around flow activities and how they may influence active game design.

### 2.4. Data collection and analysis

Delphi surveys are conducted in successive rounds until an agreement is reached or until it is clear there will be no agreement, e.g., responses are unchanged between rounds. Items for which agreement is reached are retired or removed from the survey for successive rounds. Thus, in each round, the survey becomes shorter. In this study, the Delphi went two rounds.

The criterion for removing statements from the survey was based on pooling the agree/strongly agree and the disagree/strongly disagree responses. If 70% of the respondents in Round 1 were in agreement, the item was retired, meaning the respondents were not asked the question again. Round 2 had more responses, and the questions were retired at 67% of the total or six out of nine in agreement. At the end of the second and final round of this study, only one statement still did not meet the criterion for “agreement.” The statement is described below in the analysis.

These findings are consistent with the Delphi methodology, as it is rarely seen that a Delphi is distributed more than three rounds (17, 19).

In this study, participants were given the first survey and given 2 weeks to complete it. At the end of that round, Likert responses for each item were tallied. For items that would be included in the second round of questions because consensus was not reached, the open-ended responses were examined. Quotes from the comments box that seems to be most representative of the responses were identified, and a quote or two from each side (agree or disagree) were included so that the participants may have a balanced perspective from which to reflect upon in the second round survey.

## 3. Research findings and discussion

Research question: Which, if any, game mechanics and features can a panel of experts in academia, health and the game industry agree on as valuable and impactful to the construction of successful and engaging active games?

The sample ended up being predominantly academic and practitioners and not as mixed as anticipated, so the researcher was unable to address how they varied by sector; however, even with the imbalance of groups, there was disagreement.

### 3.1. Survey

There were three main sections in the survey: (1) design regarding motivation, (2) design regarding social influences, and (3) design regarding flow.

#### 3.1.1. Findings on active game design: motivation

**Figure 1:**
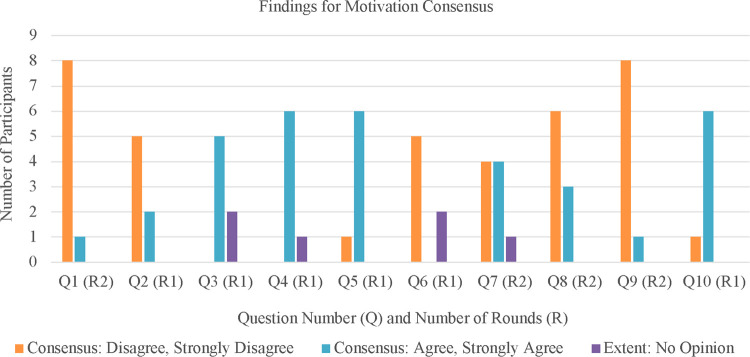
Accumulated results of the motivation questions.

There were 10 original statements asking expert panelists to consider how motivation may influence active game design. For a breakdown of statements and consensus, please refer to Supplementary Table S1. See Figure 1 for quantitative visuals on findings for motivation design consensus.

##### 3.1.1.1. Avatars and body shape

The statement offered for consensus is as follows:

• Avatars should realistically represent the player's body type, shape, weight, and height—including changing over time as the player's body change

Although consensus in the first round was not reached, there were some agreements in the comments, with one person noting the importance of giving the player control over how to create their avatar. “*The wonderful world of digital games is that the player has the ability to control their images and be who and what they want to be in the digital space*”—S8. Two respondents noted the importance of showing an avatar change over time (as the player changes). Two noted that an idealized version of the player could be good but that there could be harm in showing negative change (weight gain). In the second round, there was greater emphasis on the positive value of showing an idealized avatar (with four respondents commenting on this), as well as the importance of giving the player control over what kind of avatar to create, possibly creating one that is similar or different to self. “*I think it depends on the player's preference, whatever motivates them to play the game*”—S7 and “*Creating an idealized version of self is one of the primary reasons players enjoy avatar-based games*”—S2.

Overall, the strongest themes that emerged were that idealized versions are most accepted and control belongs to the players. Based on experts’ feedback, avatars may not need to be realistic or change over time. Players should have control of their avatars and may want an idealized version of themselves, but realistic representation is not necessary. It might even be discouraging when showing physical effects like weight changes.

##### 3.1.1.2. Feedback in active games

The statement offered for consensus is as follows:

• Feedback in active games should always be positive, thoughtful, and encouraging without criticism.

Consensus was met in the first round, with the majority (five of seven) disagreeing with the statement. Collective themes emerged; feedback should be balanced, positive, and constructive when negative. Balanced feedback is necessary as both negative and positive feedback should be distributed with tact and thoughtfulness in mind. One respondent added, “*honest direct feedback given in a thoughtful manner was key*”—S6, and “*failure to inform the players could cause misunderstandings of real-life situations resulting in failure*”—S8. Feedback is a critical component of any game, and it is particularly important in active games because it encourages the player to continue engagement in physical activity, helping them understand their progress. In-game mechanics can be used to push players to do more, set higher goals, and encourage progression. Players are allowed to create goals tailored to their specific needs.

##### 3.1.1.3. Game challenge

The statement offered for consensus is as follows:

• Game characters in-game should push players to do more and set higher goals as a way of encouraging continued progress towards the next goal.

Consensus reached in the first round with five of seven overall themes in comments is that characters are seen as motivating and may help sustain interest. One respondent stated that it could “*push people a bit, give hints and unlock for momentum*”—S3. Another respondent added, “*levels in games engage players, help sustain game play, but also allows players to understand active participation*”—S8. “*Goals should be tailored to the individuals’ current condition, as well as likes and dislikes*”—S2. It is important for developers to help players maintain a level of commitment; this can be done in a variety of ways, but respondents agree that for active games, in-game character-led motivation may be useful.

##### 3.1.1.4. Personal connection

The statement offered for consensus is as follows:

• Active games should engage with the player on a personal level using information for welcoming back players by name, maintaining friendly dialog, or making personal suggestions.

Consensus was reached, with six of seven participants agreeing or strongly agreeing with the statement. Participants’ written responses had overarching themes that personalization can be a nice choice, be unobtrusive, and not a necessary feature. Written responses were overall in agreement that personalization is a nice feature but stated that personalization should be limited. “*Introducing a personal connection and personal identifier may create a novel experience for the player, use caution as more advanced players may find this intrusive and obstructive to their game play*”—S8. Two responses indicated personalization would help engage the player. “*Making it personalized will engage the user more*”—S6. Three other participants felt it was a nice feature but not necessarily crucial. Personalization within active games includes ways in which the game tailors the activity to the player. It may include addressing the player by name and remembering returning players. This personalization can be a nice feature that may help engage the player but should be optional as it may be seen as intrusive or obstructive to gameplay.

##### 3.1.1.5. Player achievements

The statement offered for consensus is as follows:

• Active games should offer player achievements such as unlocking levels, generating scores, and leader boards.

Most of the respondents (six of seven) agreed that adding achievements to active games would be beneficial, with only one outlier in the disagree category. One respondent noted that achievements were common practice in active games, while another disagreed, stating, “*it seemed to be missing in most games and would certainly be a novel addition to active games*”—S8. While responses were fairly similar, one in particular stood out, “*tying score to physiological parameters such as heart rate*”—S8. Player achievements such as unlocking levels, generating scores, and leader boards are standard in all games, but introducing new ways to measure progress could generate continued interest.

##### 3.1.1.6. Exercise based

The statement offered for consensus is as follows:

• Active games should hide the fact that they are exercise-based.

Consensus was reached with five of seven respondents on this statement regarding the value of hiding the fact that active games include exercise, agreeing that there is no need to hide the exercise. Comments included, “*players need to be self-aware*”—S5 “*or they’ll figure it out*”—S3. In contrast, one participant did note, “*it may be useful for individuals with limited interest in exercise or children who enjoy playing in traditional games like tag*”—S8. One respondent felt it would be dependent on the situation as some kids enjoy active play while other individuals actually like exercising. Although most of these particular responses were brief, experts agreed that even though players may not find exercise interesting, it is unnecessary to hide the exercise within gameplay.

##### 3.1.1.7. Physical activity and healthy attitudes

The statement offered for consensus is as follows:

• Active games should make the activity apparent so that players can learn and acquire healthy exercise attitudes.

A majority criterion was not met in either round, making this the only question to have remained split. The comments provided to participants in Round 2 include the following:

• Agree: Better to be upfront with the user.
• Agree: Health behaviors should be positive, and we should learn to measure them.
• Agree: Ideally, the game is a gateway to an overall healthy lifestyle.

In Round 1, a respondent called it out as a poor question. Analysis of the open-ended responses reveals a split. In the first round, two noted that it depends on the situation of the player or the type of game. For example, “*an older person in rehab may not mind a health and finessed focused game, but a child would not be interested*”—S8. Four others were in agreement. One respondent felt it was better to be upfront with the players. Another recognized active games as a possible gateway to a healthier lifestyle. “*Ideally it is a gateway to healthy lifestyle*”—S3. Also, noted was, “*healthy behaviors should be positive and measured*”—S5.

In the second round, three of the four respondents agreed with the statement commenting that “*physical activity be made apparent*”—S9 and “*that it's important to foster positive messages about healthy behaviors*”—S5. Two maintained that active games be a gateway to a healthier lifestyle. The five respondents who disagreed offered that it was unnecessary to have physical activity at the forefront; it was more important to have fun, as people are already blasted with educational messages about health and exercise. One respondent with a differing view added, “*the stealthier the message, the more opportunity there is for fun to take center stage*”—S2.

The strongest themes that emerged were viewing active games as gateways to healthier lifestyles, and exercise does not have to be blatantly apparent, nor does it have to be hidden. Experts in this study prefer that players be self-aware because it teaches healthy pathways, and the real emphasis should be on fun activities that encourage positive behavior changes.

##### 3.1.1.8. Quests and storylines

The statement offered for consensus is as follows:

• Active games should embed workout activities in quests or storylines.

Consensus was not reached in the first round, so this question was asked twice to the participants, once in Round 1 and once in Round 2. Comments for consideration from Round 1 were provided in the second round and were made so based on opposing views, including

• Agree: Narratives have been used as a way to initiate and sustain interest. While active games have created a buzz and interest, sustainability has been an issue. This is similar to the stealth approach above and, as mentioned, needs to be addressed according to the target population.
• Disagree: Dance Dance Revolution or EyeToy Kinetic never had narratives, *per se*.

While consensus was not reached in the first round, there were some agreements among the respondents. Two respondents were in favor of storyline stating, “*that narratives are way to initiate and sustain interest and suggested more research in narrative medicine*”—S5 and S8. Although, one respondent disagreed, pointing out, “*Dance Dance Revolution and EyeToy Connect never had narratives*”—S7. Another favorable response mentioned the idea of having challenges interspersed in the game. Three respondents who selected *no opinion*, but left comments, stated, “that it is depended on the goals and context of the game”—S4, or “*it could go either way. I don't agree or disagree*”—S3. While the third respondent noted that “*quests were good, but was not in favor of story driven games because they distract players*”—S2.

Round 2 consensuses with six of nine participants disagreeing with a quest- or story-based approach. It seems as although the narrative piqued the interest of some participants and it is not something that is very common in active gaming, a continuing narrative might keep players coming back. However, experts cannot reach a consensus on this. The strongest theme that occurred was in favor of narrative or preferably quest-based games to sustain interest. Yet, some accompanying opinions in favor also stated they are not necessary but certainly could be a choice. The next theme that emerged was when to use such a game, and it would be dependent on goals, the context of the game, and player situation. Embedding workout activities within in-depth quests or storylines may be a new avenue to explore, as these types of games are not common in active games.

##### 3.1.1.9. Predefined goals and BMI

The statement offered for consensus is as follows:

• Goals should be set by the game for the player once BMI measurements are acquired.

A majority opinion was not reached during the first round; four respondents either *disagreed* or *strongly disagreed*, while three chose *no opinion*. Four indicated that body mass index (BMI) is an inconsistent measure and a term that turns people off. Three noted that it does not measure health and fitness and is not a good way to measure risk or change. “*BMIs are an inconsistent base measurement of health or fitness*”—S7. Two of the three respondents with *no opinion* commented that “*it depends on the context and an unnecessary feature*”—S4. Also, “*goals should be tailored to the player, as BMI is not associated with all active games*”—S2.

During the second round, the majority of the responses (eight of nine) were either *disagree* or *strongly disagree*. Written statements from six of the respondents’ noted BMI is only a single factor being measured, making it both too limiting and not accurate enough to create helpful goals for the players. Three of these respondents mentioned that goals should be set for physical activity, not weight loss. “*Goals should be set on this physical activity behavior itself, not the outcome of weight loss*”—S7.

Overall, the strongest themes that appeared were predefined goals based on BMI measurements that can be inconsistent and does not measure health and fitness levels appropriately and are too limiting of a factor to be considered reliable for players. Being cognizant of the health and fitness levels of players allows for control to be placed in the hands of the player should they prefer. This means that developers must find a variety of different factors to base players’ goals on to tailor them accurately to be most effective for the individual.

##### 3.1.1.10. Player control

The statement offered for consensus is as follows:

• The player must be able to modify goals and level of difficulty of intensity.

Six of seven respondents were in agreement that players must be able to modify goals and level of difficulty of intensity. The overall comparison was in favor of player control. One respondent in agreement mentioned that “*giving players the ability to modify aspects of the game gives them a continued sense of control and allows those with limitations to specialize the game to their needs*”—S8. The other responses from within agreement mentioned that “*goals are often not accomplished for various reasons, so the game could review uncompleted goals and offer solutions*”—S2. The respondent who disagreed noted that it depends on the context, and this feature is not required but could be beneficial. Allowing players to have control may also stimulate engagement. Player control of goals and levels of difficulty allows players to feel empowered and may lead to the completion of goals often not met due to difficulty and limitations.

#### 3.1.2. Recommendations based on feedback regarding motivation

Give players **control of their avatars**. The avatars do not have to be realistic or be perfectly rendered. As learned from Rounds 1 and 2, it is more important to let the players decide what their avatar should look like and allow the players to change it over time. “*Many play games and enjoy playing due to the virtual world which allows one to connect with characters that are different from their own identity*”—S6. Experts did not feel that making the avatar change automatically is necessary and may do harm (such as physical changes that reflect weight change), but they agree that the player should have control over their own avatar.

**Provide balanced feedback**, both positive and negative. Be tactful and thoughtful in helping the player feel positive about their play experience. Another impression taken from the Delphi methodology specifically for active games was that “*failure to provide any form of feedback that would inform users of consequences to actions may result in users misunderstanding of real life situations that do result in failure*”—S8. Do not berate the player, yell at the player, or try to motivate a player by making them feel bad about themselves.

Players measure their progress in games through scores and other metrics. The Delphi methodology revealed while these are standard game features, they are often missing in active games. “***Consider other methods of scoring such as using physiological measures as a metric****, such as heart rate change*”—S8. Sharing scores and outputs on social media could be motivational to players but should be optional.

While not necessary, it can be a nice feature to **allow a player to make the game their own**, such as calling the player by name and reflecting avatar choice. Further suggestion from the Delphi methodology was to “use personalization with caution, as more advanced players may find this intrusive to their gameplay”—S8. Give players the option to turn this off. Personalized connections can be a novel experience for the player and encourage engagement.

Some developers may try to “hide” physical activity from the player in the design of their games. Experts within the Delphi study agree this is unnecessary. “***Players should be self-aware, and recognize the importance of physical activity and healthy paths***”—S3 and S5. Players should not hurt themselves through inappropriate technique or exertion. Games can increase engagement and enjoyment of physical activity and can also help the player value a healthy lifestyle. Do not try to distract the player from this important realization.

Experts caution **against making recommendations to the player based on BMI**, as it is an inconsistent measurement and does not measure fitness levels appropriately. Give players control of goal setting and provide different levels of difficulty so that they can find the right level of exertion for each own condition.

#### 3.1.3. Findings on active game design: social influences

**Figure 2:**
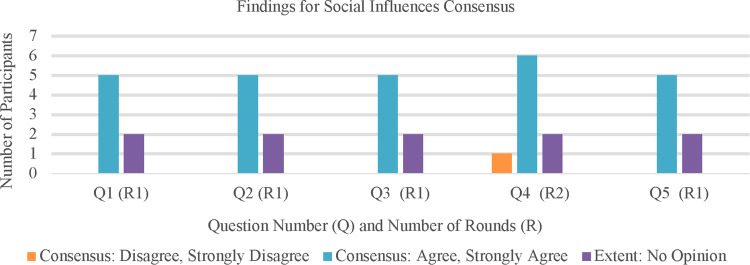
Accumulated results of the social influences questions.

There were five original statements in this section asking expert panelists to consider how social influences impact active game design in Round 1. One statement was returned to the panelists for further inquiry in Round 2. For a breakdown of statements and consensus, please refer to Supplementary Table S2. See Figure 2 for quantitative visuals on findings for social influences consensus.

##### 3.1.3.1. Social media and sharing

The statement offered for consensus is as follows:

• Active games should allow players to share progress with others *via* social media apps such as Twitter, Facebook, and Instagram.

Out of seven responses, five agreed that the ability to share information *via* social media should be an option and may be beneficial. Respondents felt that it might help validate players’ actions and, in doing so, motivate them to continue working to reach goals. “*Social media influences behavior and should be used as an alternative in a secure setting*”—S8. Interestingly, multiple respondents also noted that it should be an option to share, but not default, because it could be seen as an infringement on the players’ privacy. Two respondents with *no opinion* but chose to comment said, “*it depends on the game and the context, adding in that it is not a necessary feature, but it could be effective*”—S2 and S4.

##### 3.1.3.2. Cooperative play

The statement offered for consensus is as follows:

• Active games should support cooperative play options.

A majority of participants (five of seven) agreed or strongly agreed with the statement. The majority agreed that games should indeed support cooperative play. “*There is plethora of evidence to support the impact of engaging in exercise that include a social component*”—S8. However, others also added that it should be an option for players to have and not required. As one respondent said, games “*are better as personal journeys rather than multi-player competitions*”—S2. Active games should support the option for cooperative play, as social components can be beneficial depending on the context of the game.

##### 3.1.3.3. Competition

The statement offered for consensus is as follows:

• Active games should support competitive play options.

The majority of respondents (five of seven) agreed that actives games should offer competitive play. One respondent stated, “*competition is not absolutely necessary*”—S4, but agreed with two others that it has the possibility of increasing motivation of players. Another respondent noted, “*active gaming was a good place to incorporate learning about competition in a healthy controlled environment*”—S8. One responded with “*it depends on the game*”—S2. Deciding on how competition should be introduced into a game, or even withheld, would be dependent on the game itself and would take into consideration how it would either enhance or inhibit the overarching themes and goals of the game. As with cooperative play, competitive play can be effective in motivating people in a healthy, controlled environment.

##### 3.1.3.4. Social accountability

The statement offered for consensus is as follows:

• Social accountability in active games, through sharing goals with others and posting daily progress, makes players work harder.

The consensus was not reached in the first round, so this question was asked twice. Comments offered included in the second round include the following:

• Agree: Not always, but framed effectively, could be a greater incentive than not having any social accountability.
• Disagree: Yes and no … depends on the person.
• Agree: Some are intrinsically motivated, but this will help many.

Two participants agreed that social accountability might be a great incentive. Three respondents indicated that sharing through social media is a personal choice; for some, it may be a motivator, but for others, it may not be. “*This works for some players but not all players*”—S4. In Round 2, the majority of the respondents (six of nine) agreed that it could be effective (as compared to reflection that comes from journaling), but it should be optional. “*It varies, some this will help and others no*”—S9. Five respondents noted that it depends on the person to want to use social media as an accountability outlet. “*Some people will choose not to play the game if they are forced to post their daily progress or if the game posts their progress automatically*”—S1. One response was particularly interesting by taking a cultural perspective into consideration, whether a country's culture valued cooperation over competition. Should cooperation be of more value, players would then more likely be inclined to share.

The strongest themes to emerge suggested that social accountability is dependent upon the person, and it should be an optional feature. Experts indicated that sharing active game progress *via* social media should be an option, as it may help validate players’ actions while increasing accountability and motivation to maintain engagement with the game. Social accountability has the potential to make players work harder because their progress is being made public; the effectiveness of this would be dependent on the personal choice to share.

##### 3.1.3.5. Community

The statement offered for consensus is as follows:

• Active game developers should build a community around multiplayer active games.

Consensus was reached with a majority opinion, with five of seven participants agreeing or strongly agreeing with the statement. Creating a community was a favorable approach to engagement and adherence for the majority of respondents but not a requirement. Most notably, a respondent stated, “*building a sense of belonging to a bigger group and identifying with others with common interest is a motivator for being active and therefore would be a novel addition to the active gaming space*”—S8. Two respondents felt that community was “*dependent on the type of the game and context that it wasn't a necessary feature, but it could be effective in some games*”—S2 and S4. Providing a communal space in multiplayer games is ideal because communities create a sense of belonging and can lead to adherence and engagement.

#### 3.1.4. Recommendations based on feedback regarding social influences

Provide an option for **players to share their progress through social media**. It can help validate the player's actions and motivate them to continue playing as means of accountability. “*Some people feel validated when sharing their progress in a social platform … this may lead to further motivation and accountability*”—S7. Sharing was likened to another accountability effort of journaling, and it is seen as positive for sustaining engagement.

**Consider cooperative play spaces** as part of your design. According to the study, while cooperative play is not a necessity, working toward collaborative goals can bring a different kind of motivation: players compete against a goal rather than against another person. “*There is plethora of evidence to support the impact of engaging in exercise that include a social component*”—S8.

Your design might benefit from **using competition to enhance the effort**. Additionally, the Delphi methodology reiterated that competition is an apparent aspect of our lives in sports, exercise, and living. “*Most of the time options for competition seem to enhance effort. There is a place for non-competitive as well*”—S3. However, traditional exercise often includes a competitive component; transferring that to gameplay in a controlled environment can introduce a healthy understanding of competitive behaviors.

**Establish a place for community engagement**. The study showed that creating a community was a favorable approach to engagement and adherence. “*Building a sense of belonging to a bigger group and identifying with others with common interest is a motivator for being active*”—S8. It does not have to be in the form of multiplayer games but rather a space for networking. When given the option to identify with a group, players can build a sense of belonging with people of common interest, such as choosing to be active through gameplay.

#### 3.1.5. Findings on active game design: flow

**Figure 3:**
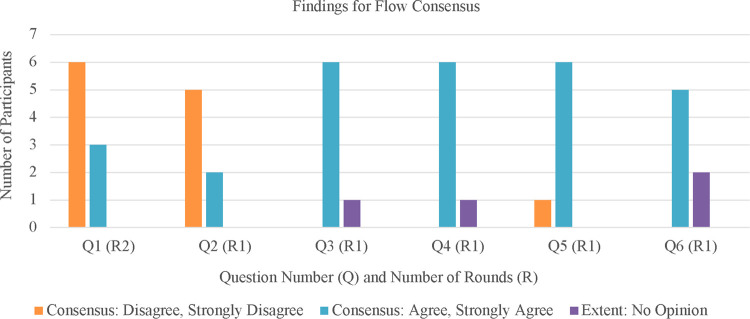
Accumulated results of the flow questions.

There were six original statements in this section asking expert panelists to consider how *flow* may influence active game design in Round 1. One statement was returned to the panelists for further inquiry in Round 2. For a breakdown of statements and consensus, please refer to Supplementary Table S3. See Figure 3 for quantitative visuals on findings for flow consensus.

##### 3.1.5.1. Achieving flow

The statement offered for consensus is as follows:

• For active games, a primary goal is to associate the desirable “flow” state with exercising, not with gaming.

Consensus was not reached in the first round, with three respondents offering “no opinion” and others equally split between disagree and agree options. Responses within the first round were quite mixed. One noted that gaming and exercise produced flow, and the combination might very well achieve a flow state. Another respondent agreed but said, “*but not every day will bring nirvana*”—S3. Two respondents disagreed, stating it was unrealistic and disruptive to achieve a flow state. Those with responses in the *no opinion* category remarked it was dependent on the type of game, while the other said, “*gaming endurance and exercise endurance are not mutually exclusive and may not stimulate the same triggers*”—S7.

The second round saw more in accordance with four respondents agreeing that flow and exercise go together and are important for a full gaming experience. “*With and active game the two go hand and hand. It is the total experience of the activity that should be consider ‘flow’. If one does not exist with the other you can not reach a flow state*”—S4. Two indicated that while flow is nice to achieve, it is not necessary. “*It is not bad to just exercise for the purpose of exercising. It is nice to be able to focus on the flow, but not crucial for all games*”—S9. The majority of comments were in agreement that exercise and gaming should not be viewed independently of one another, and they are dependent on each other for the player to reach a flow state. Creating opportunities for flow in active games can prove to be challenging, as there is the flow of gameplay and the flow of exercise. Trying to associate the “flow” state with one and not the other is not advised. Flow is important to both and should go hand and hand with one another; a balance is needed to fulfill a flow state.

##### 3.1.5.2. Achieving flow and personal goals

The statement offered for consensus is as follows:

• To help players achieve “flow,” designers should make players set overall personal goals such as losing weight, running faster, or achieving personal best.

A majority opinion (five of seven) sided with disagree and strongly disagree that goal setting is not necessary to not help achieve flow. “*Goals may not be properly matched with skill set to meet the challenge which would negate the possibility of connecting with the higher flow state*”—S8. Two respondents in this group added, “*that flow is a very intuitive process, while goal setting is cognitive*”—S5. Requiring players to set personal physical goals, such as losing weight, running faster, or achieving personal best, is not ideal because the expectations of meeting set goals may not match the player's skill set, negating induced flow.

##### 3.1.5.3. Controlling exercise and routines

The statement offered for consensus is as follows:

• Active games should allow players to cultivate chances for enjoyment, for example, mixing and matching exercises or creating their routines.

The consensus was reached, with six of seven participants agreeing or strongly agreeing. Collectively themes such as ownership and flexibility appeared. Two respondents strongly agreed, and one said, “*allowing player flexibility is great way to take ownership, it demonstrates learning, which will lead to higher levels of efficacy*”—S7. Another respondent stated, “*personalization and allowing players to choose would stimulate interest and motivate them to continue playing*”—S8. Two more stated that flexibility could help, but it is “*likely not critical, and it depends on the context of the game*”—S3 and S4. Allowing players the flexibility to mix and match exercises or to create their routines helps create a sense of ownership and demonstrates learning, which will lead to higher levels of efficacy.

##### 3.1.5.4. Performance feedback

The statement offered for consensus is as follows:

• Active games should provide players with information about their performance during play.

A majority of respondents (six of nine) were in agreement or strong agreement with the statement. Motivation and the necessity for feedback ended up being the strongest themes. Although responses to this question were limited, one respondent said, “*the choice would be dependent on the player and the situation, because an advanced player may find the constant interrupt annoying while a novice might find it interested and stimulating*”—S8. The other three respondents noted that feedback is usually “*motivating, it is necessary and informs them of performance such as not being able to pass a level*”—S6, S5, and S3. “*Active games should give players information about their performance while they are playing*.” This feedback is helpful and motivates the players to continue when it is strategically placed in the game.

##### 3.1.5.5. Increasing difficulty

The statement offered for consensus is as follows:

• As players concentrate harder and continue to acquire skills, gameplay should become increasingly difficult.

A majority of participants (six of seven) agreed or strongly agreed. Themes appear to relate to a sense of accomplishment, and activities need to be aligned with a player's skill level. There were two who strongly agreed but left out comments. Three respondents noted that level progression keeps players engaged and allows them to feel a sense of accomplishment. Additionally, “*it provides players with the opportunity to engage at their skill level in which they may reach a flow state*”—S8. Another respondent said, “*balance was essential and maintaining a steady performance was okay*”—S3. The respondent who disagreed stated, “*exercise adherence isn't about the exercise getting harder, it's about coordinating game progression with basic fitness principles such as, frequency, intensity, time and duration. Increasing volume should take priority over difficulty*”—S8. As players advance during gameplay, so should the difficulty, which should be driven by levels of exercise such as frequency, intensity, and duration in consideration of a player's abilities.

##### 3.1.5.6. Predetermined challenge levels

The statement offered for consensus is as follows:

• Active games should help players choose challenging levels of play.

Consensus was reached with (five of seven) participants in agreement: games should help players find the right level of challenge. The overarching idea that emerged was that players need to have a choice. Providing support, but not taking away the player's choice for selecting challenging gameplay, was noted by four of the respondents. One further commented, “*while providing support, further investigation would be necessary as support may also be a distractor for advanced players*”—S8. The one *no opinion* responder who provided feedback said, “*it depends on the game as sometimes help is perceived as coddling and hand-holding, which is a turnoff for many players*”—S2. The second *no opinion* responder felt that they needed more of an explanation of what the researcher meant by “helping” the player. Helping players choose challenging levels to play works if the player still has some choice, as providing too much guidance may prove distracting.

#### 3.1.6. Recommendations based on feedback regarding flow

When players enter an effortless state of engagement and reach an optimal level of concentration, they are happiest. The world around them ceases to exist when they are deeply absorbed in their activity. Results of this survey indicated that **there is room for exploration, but this may be the most difficult area within the design process**, but not impossible.

Design activities that are not overly difficult or mundane to perform. When combined with gaming, the ideal would be an **achievable activity that is well balanced with gameplay** according to what we learned from both rounds of the Delphi study. “*With an active game the two go hand and hand. It is the total experience of the activity that should be consider flow*”—S4. The more conscious the player is of the physical activity, the shorter duration of play. Additionally, experts felt that the two areas, gaming and physical activity, should be thought of as a unit and not separately, but that flow will not always be achievable for both.

**Grant the player the flexibility to modify the physical activity** they would like to engage in. The experts from the study suggest that adjustability will allow the player to take ownership; it may engage and motivate them. “*Personalizing and allowing players to choose options within the game could stimulate interest and motivate players to continue playing as they choose their exercise and routines*”—S8. “*Also, it demonstrates a higher level of learning and efficacy*”—S7.

**Integrate game progression and basic fitness principles** such as frequency, intensity, time, and duration. The Delphi study revealed that **exercise adherence is n**o**t about physical activity getting harder** and progression is an opportunity to engage in activities consistent with skill. “*Increasing volume of frequency, intensity, time and type/duration should take priority over difficulty*”—S7. Create opportunities such as leveling up for players to engage in activities suited to their abilities rather than increasing game difficulty.

### 3.2. Discussion

This study asked, “Which, if any, game mechanics and features can a panel of experts in academia, health, and the game industry agree on as valuable and impactful to the construction of successful and engaging active games?” The experts were able to reach an agreement on 20 of the 21 questions.

#### 3.2.1. Limitations

This study would have been strengthened with the inclusion of more game designers. While the initial list of participants included a balanced list of game designers, practitioners, and academics, the request to participate was only answered by one game designer. Additionally, the study would have benefitted from a larger number of expert participants. Because of the specific timeline for the study, seeking additional participants became an issue as this particular field has a limited number of known experts. Once the request to participate was sent to the select group, the 1-week period for responding began.

There were unanticipated challenges once the survey was underway. The participants remained anonymous through the entire study process; however, coding of actual subjects at the beginning of the study would have enabled reliable tracking for both rounds. Not having done so resulted in all of the original agreeing participants being emailed again in the second announcement.

#### 3.2.2. Considerations for future research

The purpose of this study was to define game design strategies for the development of active games. As technology continues to advance and newer active games become available, a larger-scale Delphi study similar to this one could be beneficial. This field continues to grow, and there will be a larger pool of experts from which to choose. The study should include a balanced group of experts with an equal number of participants from each field.

Most professional game developers keep up with technology trends and understand the intricacies of design. These perspectives are invaluable to the development of enhanced active games. Had there been more game designers in this study, there may have been more clarity within the comments sections regarding design, thus providing a different perspective for the researchers and practitioners. Likewise, it would have been beneficial for game designers to gain insight from those who actually put active games into practice.

Interestingly, the remaining question also provides an opportunity to examine the split more thoroughly. A study examining whether there are behavioral changes as a result of playing active games both for physical fitness and pure leisure that are using current technologies is needed. A similar one could study the other side of the split and examine the engagement and sustainability of active games, for example, those that are designed explicitly for physical fitness and those created solely for play.

Another qualitative study for consideration would be case studies with actual active game players. While it was very informative to gain perspective from experts who research and implement active game spaces, it is very important not to dismiss the gamers themselves. While this study was based on a more theoretical perspective of engagement, studying gamers who are actually active participants in physical games is extremely important. The researcher will gain first-hand knowledge of what the users perceive as motivating and how flow states are reached, and a better understanding of social influences should one conduct a similar study.

Additional follow-up studies can be conducted in the areas of augmented and virtual reality (VR) for active gaming. While VR technology is not in the majority of homes just yet, it is becoming more affordable and is now more readily available to consumers. More applications are being built and implemented fairly consistently now. There may be an increased sense of realism that could be a very interesting perspective to study, especially with physically enhanced gaming.

Another area for consideration is creating active games for mobile devices. Pokémon Go was released in the summer of 2016 and, to date, is reported as the most popular augmented reality game (20). In 2020, it reached $1.23 billion in revenue (21). Due to the continued success of Pokémon Go, there has been an upswing in active game design, with similar types of products emerging for mobile devices. Smartphones are already able to track health data such as heart rate and steps taken. This could lead to more health and fitness-based studies using mobile technology.

While this study was not able to fully recommend quest-based games, this is another area for consideration, especially when games can be designed with mobile devices in mind. Realizing that narrative and quest-based games may not be enjoyed by everyone, there certainly could be a growing market for them.

Some experts in the study did find quest-based games as something worth exploring in future research, although they were not familiar with games such as Pokémon Go at the time. As our society continues to engage in social media, any of the above suggestions for future studies could incorporate how social media and communities are formed around active games and mobile technologies.

## 4. Conclusion

The field of active gaming has matured, and there are pockets of experts in design, research, and implementation. There are consistent best practices defined; however, they are not implemented in all games yet. The necessity and excitement for active gaming are still there; maintaining player enthusiasm and engagement in these types of games consistently is an issue. Through better game design and newer types of active games, a resurgence of active game players will appear. The reality is active games need to be created that incite engagement and commitment to use for extended periods of time. These guidelines can inform developers working with newer technologies such as mobile devices, enhanced gaming consoles, and virtual and augmented reality platforms to create active games that inspire gamers to play.

## Data Availability

The original contributions presented in the study are included in the article/**Supplementary Material**, further inquiries can be directed to the corresponding author.
