# Supplementary material for: Design recommendations for active games
Source: Front Digit Health. 2022 Sep 16;4:814226. doi: 10.3389/fdgth.2022.814226 (PMC9795476; doi:10.3389/fdgth.2022.814226)
Supplement: Supplementary file 1 [file Table1.docx]

Supplementary Material

Table 1. Findings for Motivation Consensus

| Statement Offered for Consensus | Consensus | Consensus | Extent | Rounds |
| --- | --- | --- | --- | --- |
|  | Disagree  Strongly Disagree | Agree Strongly Agree | No Opinion |  |
| 1. Avatars should realistically represent the player’s body type, shape, weight and height— including changing over time as the player’s body changes. | 8 | 1 | 0 | 2 |
| 2. Feedback in active games should always be positive, thoughtful and encouraging without criticism. | 5 | 2 | 0 | 1 |
| 3. Game characters in-game should push players to do more and set higher goals as a way of encouraging continued progress towards the next goal. | 0 | 5 | 2 | 1 |
| 4. Active games should engage with the player on a personal level using information for welcoming back players by name, maintaining friendly dialogue, or making personal suggestions. | 0 | 6 | 1 | 1 |
| 5. Active games should offer player achievements such as unlocking levels, generating scores and leader boards. | 1 | 6 | 0 | 1 |
| 6. Actives games should hide the fact that they are exercise based. | 5 | 0 | 2 | 1 |
| 7. Active games should make the activity apparent, so that players can learn and acquire healthy exercise attitudes. | 4 | 4 | 1 | 2 |
| 8. Active games should embed workout activities in quest or story lines. | 6 | 3 | 0 | 2 |
| 9. Goals should be set by the game for the player once BMI measurements are acquired. | 8 | 1 | 0 | 2 |
| 10. The player must be able to modify goals and level of difficulty of intensity. | 1 | 6 | 0 | 1 |

Table 2. Findings for Social Influences Consensus

| Statement Offered for Consensus | Consensus | Consensus | Extent | Rounds |
| --- | --- | --- | --- | --- |
|  | Disagree  Strongly Disagree | Agree Strongly Agree | No Opinion |  |
| 1. Active game should allow players to share progress with others via social media apps such as Twitter, Facebook and Instagram. | 0 | 5 | 2 | 1 |
| 2. Active games should support cooperative play options. | 0 | 5 | 2 | 1 |
| 3. Active games should support competitive play options. | 0 | 5 | 2 | 1 |
| 4. Social accountability in active games, through sharing goals with others and posting daily progress, makes players work harder. | 1 | 6 | 2 | 2 |
| 5. Active games developers should build community around multiplayer active games. | 0 | 5 | 2 | 1 |

Table 3. Findings for Flow Consensus

| Statement Offered for Consensus | Consensus | Consensus | Extent | Rounds |
| --- | --- | --- | --- | --- |
|  | Disagree  Strongly Disagree | Agree Strongly Agree | No Opinion |  |
| 1. For active games a primary goal is to associate the desirable ‘flow’ state with exercising, not with gaming. | 6 | 3 | 0 | 2 |
| 2. To help players achieve ‘flow' designers should make players set personal overall goals such as losing weight, running faster or personal best. | 5 | 2 | 0 | 1 |
| 3. Active games should allow players to cultivate chances for enjoyment, for example mix and matching exercise or creating their own routines. | 0 | 6 | 1 | 1 |
| 4. Active games should provide players with information about their performance during play. | 0 | 6 | 1 | 1 |
| 5. As players concentrate harder and continue to acquire skills, gameplay should become increasingly difficult. | 1 | 6 | 0 | 1 |
| 6. Active games should help players choose challenging levels of play. | 0 | 5 | 2 | 1 |
