# Supplementary material for: Design recommendations for active games
Source: Front Digit Health. 2022 Sep 16;4:814226. doi: 10.3389/fdgth.2022.814226 (PMC9795476; doi:10.3389/fdgth.2022.814226)
Supplement: Supplementary file 2 [file Image1.pdf]

Pepperdine University
24255 Pacific Coast Highway
Malibu, CA 90263
TEL: 310-506-4000

NOTICE OF APPROVAL FOR HUMAN RESEARCH

Date: March 02, 2016

Protocol Investigator Name: Pamela Martinez

Protocol #: 16-02-210

Project Title: Active Games: An Examination of User Engagement to Define Design Recommendations

School: Graduate School of Education and Psychology

Dear Pamela Martinez:

Thank you for submitting your application for exempt review to Pepperdine University's Institutional Review Board (IRB). We appreciate the work you have done on your proposal. The IRB has reviewed your submitted IRB application and all ancillary materials. Upon review, the IRB has determined that the above entitled project meets the requirements for exemption under the federal regulations 45 CFR 46.101 that govern the protections of human subjects.

Your research must be conducted according to the proposal that was submitted to the IRB. If changes to the approved protocol occur, a revised protocol must be reviewed and approved by the IRB before implementation. For any proposed changes in your research protocol, please submit an amendment to the IRB. Since your study falls under exemption, there is no requirement for continuing IRB review of your project. Please be aware that changes to your protocol may prevent the research from qualifying for exemption from 45 CFR 46.101 and require submission of a new IRB application or other materials to the IRB.

A goal of the IRB is to prevent negative occurrences during any research study. However, despite the best intent, unforeseen circumstances or events may arise during the research. If an unexpected situation or adverse event happens during your investigation, please notify the IRB as soon as possible. We will ask for a complete written explanation of the event and your written response. Other actions also may be required depending on the nature of the event. Details regarding the timeframe in which adverse events must be reported to the IRB and documenting the adverse event can be found in the *Pepperdine University Protection of Human Participants in Research: Policies and Procedures Manual* at community.pepperdine.edu/irb.

Please refer to the protocol number denoted above in all communication or correspondence related to your application and this approval. Should you have additional questions or require clarification of the contents of this letter, please contact the IRB Office. On behalf of the IRB, I wish you success in this scholarly pursuit.

Sincerely,

Judy Ho, Ph.D., IRB Chairperson

cc: Dr. Lee Kats, Vice Provost for Research and Strategic Initiatives

Pepperdine University
24255 Pacific Coast Highway
Malibu, CA 90263
TEL: 310-506-4000

Mr. Brett Leach, Regulatory Affairs Specialist
